# Supplementary material for: Single-Step Fabrication of Au-Fe-BaTiO3 Nanocomposite Thin Films Embedded with Non-Equilibrium Au-Fe Alloyed Nanostructures
Source: Nanomaterials (Basel). 2022 Oct 3;12(19):3460. doi: 10.3390/nano12193460 (PMC9565752; doi:10.3390/nano12193460)
Supplement: Supplementary file 1 [file nanomaterials-12-03460-s001.zip › nanomaterials-1924250-supplementary.pdf]

nanomaterials

Supplementary Materials

Single-Step Fabrication of Au-Fe-$BaTiO_3$ Nanocomposite Thin Films Embedded with Non-Equilibrium Au-Fe Alloyed Nanostructures

Bethany X. Rutherford [1], Hongyi Dou [1], Bruce Zhang [2], Zihao He [2], James P. Barnard [1], Robynne L. Paldi [1] and Haiyan Wang [1,2,*]

[1] School of Materials Engineering, Purdue University, West Lafayette, IN 47907, USA
[2] School of Electrical and Computer Engineering, Purdue University, West Lafayette, IN 47907, USA
* Correspondence: hwang00@purdue.edu

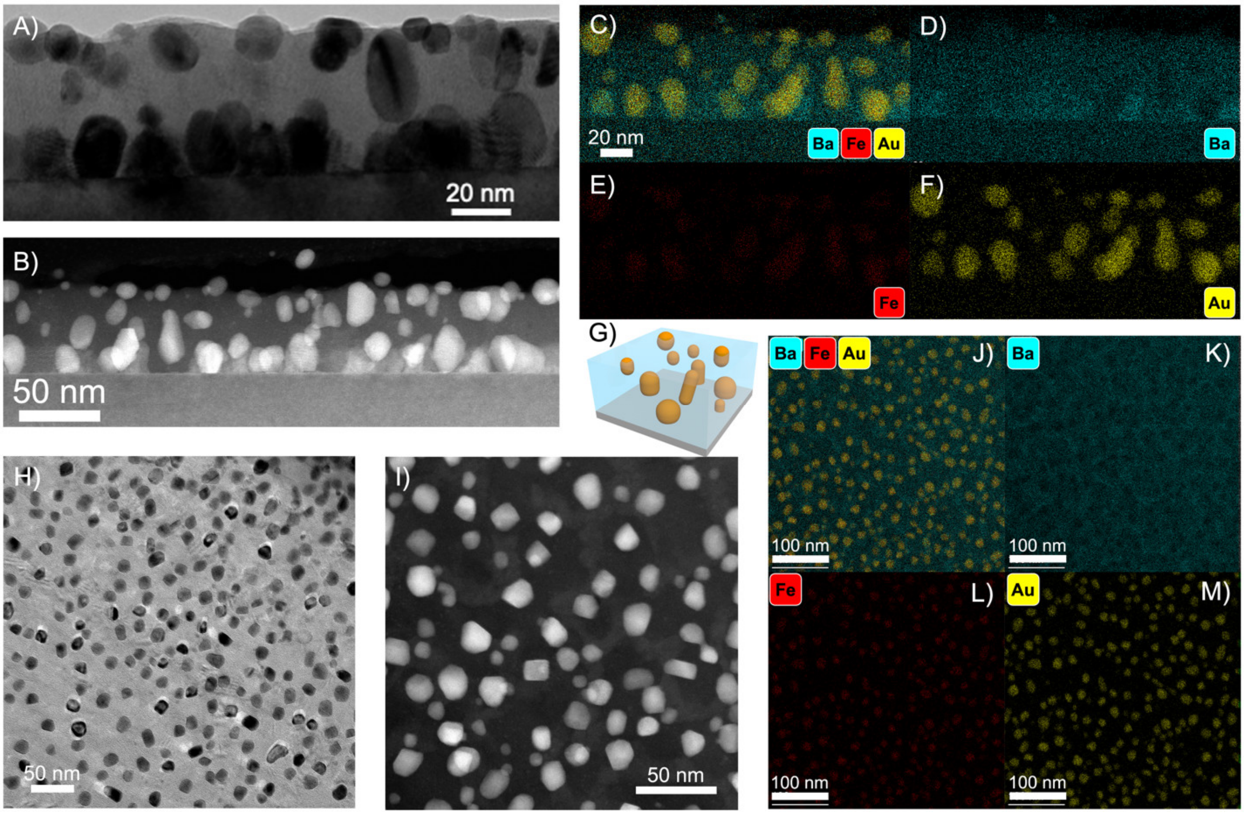

Figure S1. 10 Hz sample TEM results: (**A**) cross-section TEM image, (**B**) cross-section STEM image, (**C**) cross-section EDX composite of Ba, Fe, and Au, (**D**) cross-section EDX of Ba, (**E**) cross-section EDX of Fe, (**F**) cross-section EDX of Au, (**G**) 3D schematic of 10 Hz sample, (**H**) plan-view TEM image, (**I**) plan-view STEM image, (**J**) plan-view EDX composite of Ba, Fe, and Au, (**K**), EDX composite of Ba, (**L**) EDX composite of Fe, (**M**) and EDX composite of Au.

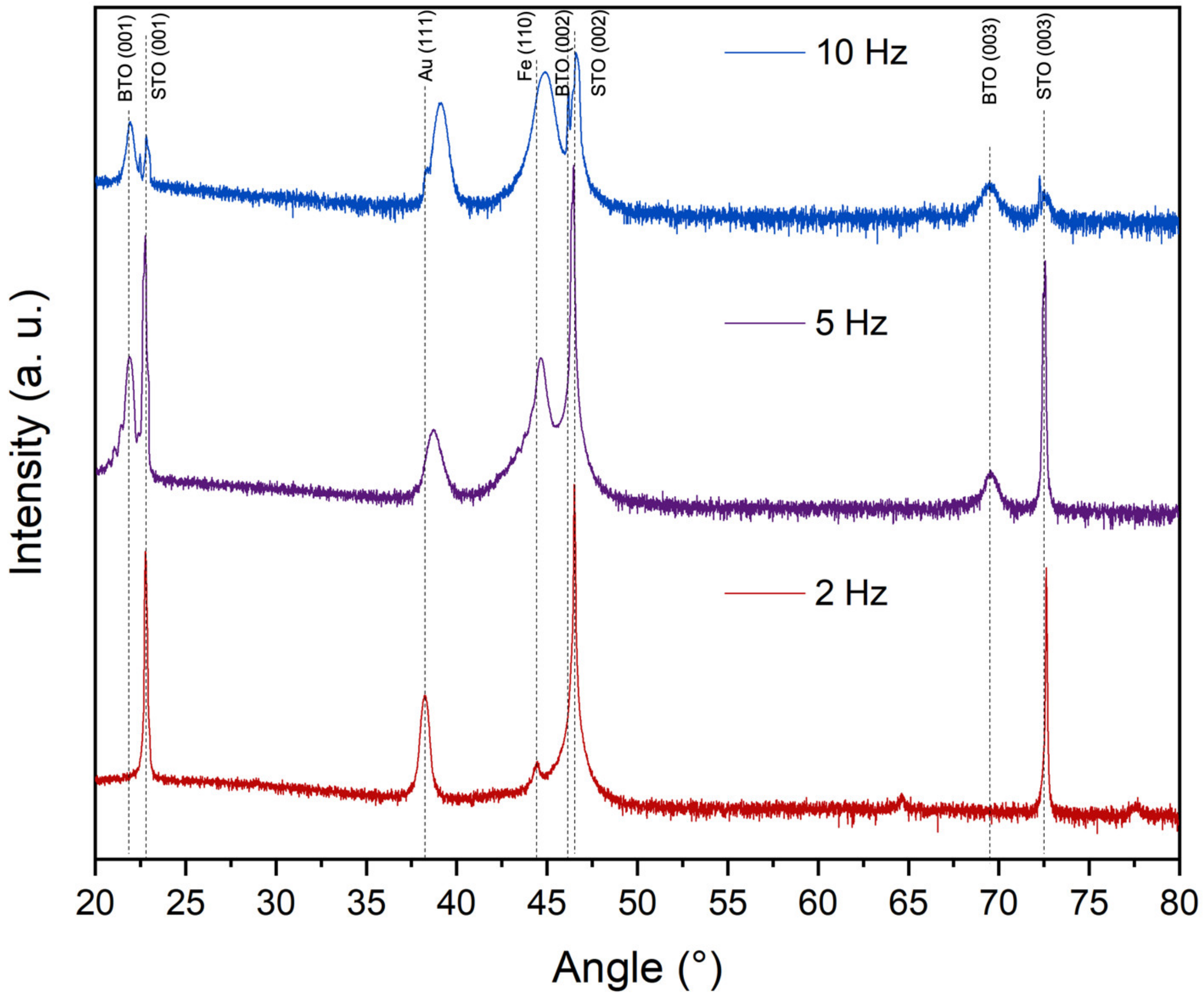

Figure S2. Full XRD spectrum for each sample.

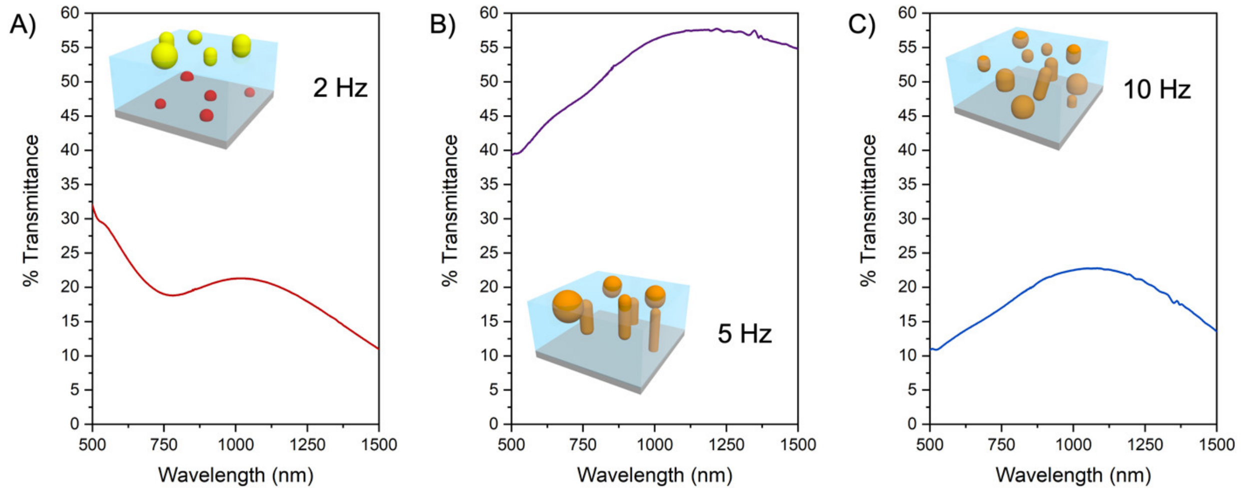

Figure S3. Optical transmittance of the Au-Fe-BTO sample grown at (**A**) 2 Hz, (**B**) 5 Hz, (**C**) and 10 Hz.
